# A feasibility study to estimate optimal rigid‐body registration using combinatorial rigid registration optimization (CORRO)

**DOI:** 10.1002/acm2.12965

**Published:** 2020-10-17

**Authors:** Afua A. Yorke, David Solis, Thomas Guerrero

**Affiliations:** ^1^ Department of Radiation Oncology UW Medicine Seattle WA USA; ^2^ Department of Radiation Oncology Beaumont Health Royal Oak MI USA; ^3^ Department of Physics Mary Bird Perkins Cancer Center Baton Rouge LA USA; ^4^ Oakland University William Beaumont School of Medicine Rochester Hills Auburn Hills MI USA

**Keywords:** central limit theorem, combinatorial rigid registration optimization (CORRO), independent trials, joint histogram, joint entropy

## Abstract

**Purpose:**

Clinical image pairs provide the most realistic test data for image registration evaluation. However, the optimal registration is unknown. Using combinatorial rigid registration optimization (CORRO) we demonstrate a method to estimate the optimal alignment for rigid‐registration of clinical image pairs.

**Methods:**

Expert selected landmark pairs were selected for each CT/CBCT image pair for six cases representing head and neck, thoracic, and pelvic anatomic regions. Combination subsets of a k number of landmark pairs (k‐combination set) were generated without repeat to form a large set of k‐combination sets (k‐set) for k = 4,8,12. The rigid transformation between the image pairs was calculated for each k‐combination set. The mean and standard deviation of these transformations were used to derive final registration for each k‐set.

**Results:**

The standard deviation of registration output decreased as the k‐size increased for all cases. The joint entropy evaluated for each k‐set of each case was smaller than those from two commercially available registration programs indicating a stronger correlation between the image pair after CORRO was used. A joint histogram plot of all three algorithms showed high correlation between them. As further proof of the efficacy of CORRO the joint entropy of each member of 30 000 k‐combination sets in k = 4 were calculated for one of the thoracic cases. The minimum joint entropy was found to exist at the estimated mean of registration indicating CORRO converges to the optimal rigid‐registration results.

**Conclusions:**

We have developed a methodology called CORRO that allows us to estimate optimal alignment for rigid‐registration of clinical image pairs using a large set landmark point. The results for the rigid‐body registration have been shown to be comparable to results from commercially available algorithms for all six cases. CORRO can serve as an excellent tool that can be used to test and validate rigid registration algorithms.

## 1 INTRODUCTION

Image guided radiation therapy (IGRT) involves imaging patients before and during radiation treatment with the aim of increasing the dose to the tumor while minimizing radiation to healthy tissues. One of the implementations of IGRT requires the ability to register the daily cone beam computed tomography images (CBCT) to the reference planning CT image set.^1^ The calculated patient position correction is then used to shift the patient into near perfect alignment with the reference image position prior to beam delivery. Image guided radiation therapy depends extensively on image registration for spatial alignment and quantitative evaluation for changes in the position and size of the target and normal tissues due to weight loss and tumor response.^2^

The registration process geometrically aligns two images by finding the minimum of an objective function representing the alignment quality (typically the mean squared error to determine the parameters of a rigid registration transformation matrix). Validation and quantification of image registration quality still poses a very challenging problem in clinical practice due to the lack of an underlying ground truth. Currently, there are four leading methods to assess registration quality: visual inspection, use of fiducials, landmark point sets, and mutual information.

Conventionally, physicians have validated registered images by visually inspecting portal images and diagnostic quality images alongside a planning digital reconstructed radiograph (DRR).^3^ This method of assessing image registration quality typically involves the use of an in‐field metric (graticule mounted to the MV treatment head) to identify anatomical structures shared between the portal image and DRR, typically the bony anatomy. The accuracy of this method has been reported to be between 5 to 10 mm.^4^ This type of registration is subjective and cannot be used for large quantities of data.^5^

Fiducial markers on phantoms and patients have also been used to find the ground truth of a rigid image registration.^6^ Due to the visibility of gold markers placed in the prostate in kV or MV x‐ray imaging, they are used to improve targeting for surgical procedures and radiotherapy treatment to the prostate and to estimate registration error.^7^, ^8^, ^9^, ^10^ Although fixed to the target anatomy, the fiducials are known to drift from the original fixed point either due to anatomy changes over time or detachment of the fiducial. The effect of fiducial relocation may lead to interobserver error associated with the registration.^11^, ^12^, ^13^ Also, the number of fiducials that can be fixed at any time is limited. O’Neill et al.^14^ described that in a study of 427 patients undergoing intensity modulated radiation therapy using fiducial marker IGRT the intrafraction motion was found to be greater than 2mm for about 66% of their patients.

Expert positioned landmark point pairs have also been used to quantitatively validate registration quality. These landmark points are used to specify the ground truth in correlated images which allows for validation based on the accuracy of the manual or automatic selection of the points.^15^, ^16^, ^17^

In this study, a metric for registration quality assessment through the development of a new approach to rigid registration we call combinatorial rigid registration optimization (CORRO). In CORRO, we generate large landmark sets from an expert selected anatomical landmark set using the mathematics of combination without replacement. We present a method for the quantitative measurement of registration quality between CORRO and commercially available rigid registration software based on mutual information and joint entropy minimization.

## 2 MATERIAL AND METHODS

### 2.A Image data

**Fig. 1:**
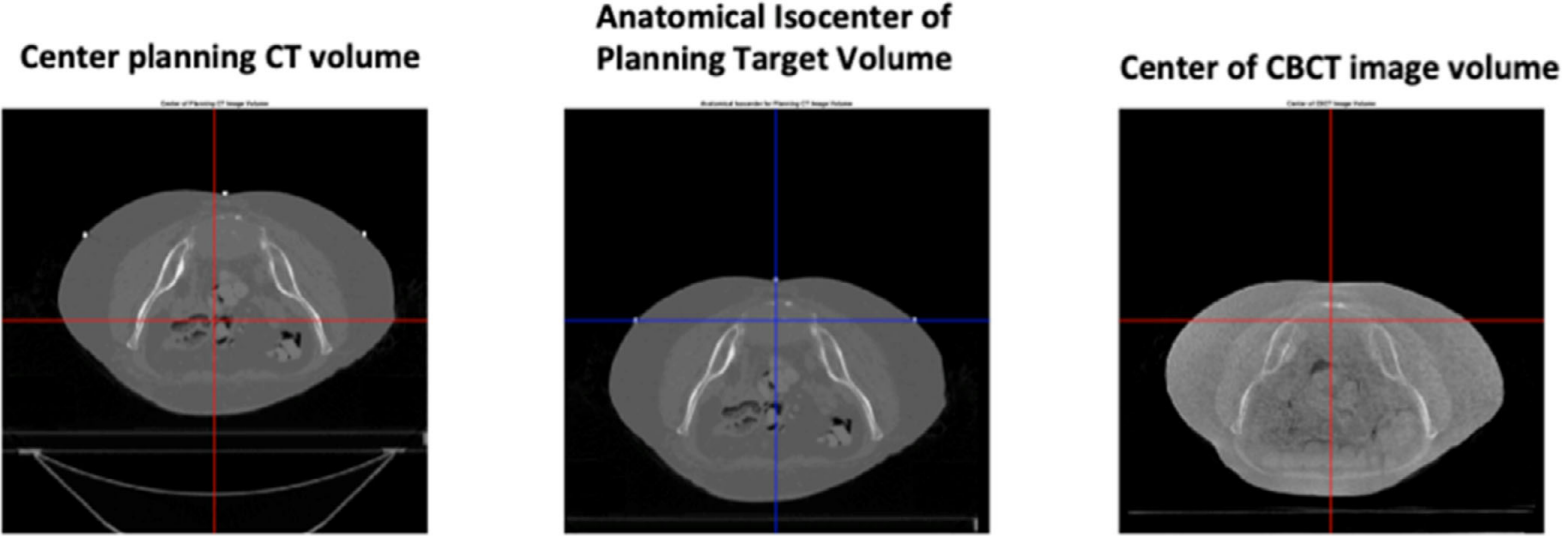
Planning computed tomography (CT) and cone beam CT images.

Six patients treated at the Beaumont Proton Center were selected for a Beaumont Research Institute Institutional Review Board approved retrospective study (2014‐326). Each patient received a planning CT on a 16‐slice Philips Brilliance Big Bore CT scanner (Philips NA Corp, Andover, MA) covering the entire anatomic region and utilizing an immobilization system. Each patient had CBCT images acquired for daily image guidance on the ProteusONE Proton therapy machine (Ion Beam Applications S.A., Belgium). The CBCT images were 768 × 768 × 110 voxel with voxel size ranging from (0.6406 × 0.6406) to (0.5176 × 0.5176) mm^2^ and 2.5 mm slice thickness for all cases. The machine isocenter is located at the center of the CBCT reconstruction image volume. The planning CT was resampled to the same dimensions in the *X*/*Y* plane as the CBCT and the image content was shifted to place the anatomic isocenter at the center of the planning target volume as shown in Fig. 1.

### 2.B Landmark pair selection

**Fig. 2:**
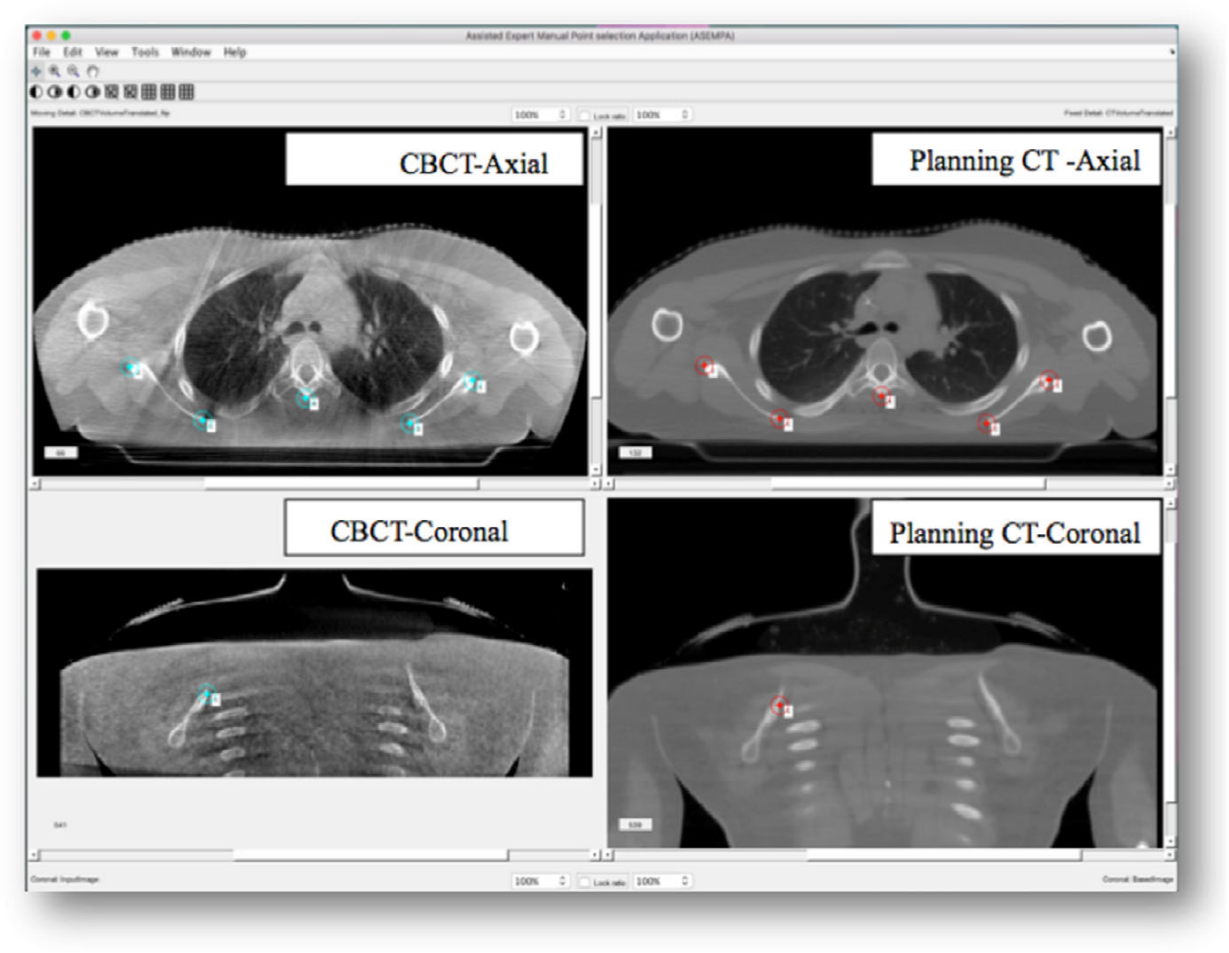
Assisted expert manual point selection application interface showing sample data. The Image on the left panel (top and bottom) shows cone beam computed tomography (CBCT) of the target image and image on the right panel (top and bottom) shows the planning CT image, which is the reference image.

An in‐house MATLAB‐based interface called Assisted Expert Manual Point Selection Application (ASEMPA) was developed to aid experts in manually selecting landmark feature pairs between the images. Figure 2 shows the main interface display. Two volumes are displayed in the axial and coronal plane. The image volume in the left panels is the CBCT and in the right panels is the planning CT.

### 2.C Rigid‐body registration

Given the CBCT (**P**) and the planning CT (**Q**), we calculate the transformation **Q = T(P)** such that the corresponding coordinates in the two images correspond to the same physical location in both images. Let $P=\left\{p_{1}\ldots p_{k}\right\}$ and $Q=\left\{q_{1}\ldots q_{k}\right\}$ denote the collection of points in $R^{3}$ with the same size, with **P** representing landmark points in the planning CBCT image and **Q** representing corresponding landmark points in the CT image. The registration problem in three‐dimension (3D) consists of finding the transformation that achieves the best match between the corresponding features in ***P*** and ***Q*** such that the root mean square (RMS) distance $d_{i}$ between corresponding points is minimized.^9^, ^18^ The appropriate translation vector is simply the mean displacement between the two sets of points.^8^ The aim is to find the errors associated with locating the landmark points. The image registration problem is reduced to a shape analysis problem or to the orthogonal Procrustes problem.^8^, ^9^, ^19^, ^20^ The Procrustes problem is simply a least square‐fitting problem and studies have shown that the calculation of the rotation matrix **R** is more involved due to the nonlinear condition for a rotation matrix to be orthogonal. If P and **Q** are replaced with their centroid values then the optimal transformation is represented as

(1) $$p_{i}\to p_{i}-\bar{p}$$

(2) $$q_{i}\to q_{i}-\bar{q}$$

This reduces the problem to the orthogonal Procrustes problem where we seek to find the rotation **R**. The RMS distance to be minimized is termed as the fiducial registration error (FRE).^8^, ^9^ Therefore, given four noncoplanar points for a 3D volume the problem of rigid‐body registration is to finding a rotation and translation (**t**) that minimizes the FRE which is represented mathematically as

(3) $${\mathrm{FRE}}^{2}\equiv \frac{1}{k}\sum_{i=1}^{k}{\left|{\mathrm{Rp}}_{i}+t-q_{i}\right|}^{2}$$

An FRE value of zero means the rigid‐body registration is perfect. However, the FRE is expected to me more than zero due to error associated with locating the landmark points.

FRE can be used to characterize the magnitude of the error associated in locating these landmark points. The translation is given by

(4) $$t=\bar{q}-R\bar{p}$$

where the bar indicates a mean over ***i = 1,…, k***.

In this study, no rotation is used though in general it could be used for robotic couch that allows for 6 degree of rotation.

### 2.D Generation of large k‐Sets

Using the anatomical landmark pairs that have been selected by expert we generate our k‐set as follows. A k‐combination set was generated as a subset of the landmark set by combining k number of landmark pairs without repeat. The set of all possible unique k‐combinations forms a large set of discrete independent trials, which we term the k‐set. In this study, three different sizes of the k‐combination set were used, k = 4, 8, 12. The mathematics is similar to the problem of determining the number of unique hands of cards from a standard deck of 52.

Let $\left\{N_{I}\right\}i=1,n$ be the locations for the landmark points selected by the expert. We find the combination without repetition of the elements such that

(5) $$\left(\begin{array}{c} N\\ k \end{array}\right)=\frac{N!}{k!\left(N-k\right)!}$$

where **k = **4, 8, 12. With this approach we find all possible independent combinations to calculate the best affine fit. The purpose of using combination without replacement is to generate millions of independent trials from a modest number of expert‐selected landmark pairs. Consider for example, if k‐combination sets of k = 4 landmark pairs are needed to solve the affine transformation, and an expert selects 48 landmark pairs, there will be 194 580 possible k‐combination sets to solve the problem. Likewise, there will be 377 348 994 combinations of k = 8 landmark pairs and 69 668 534 468 combinations for k = 12 landmark pairs. It should be noted that not every discrete independent combination would give an acceptable affine transformation, as many of the points may be collinear. The points are filtered to get the best points for registration by finding the least FRE and putting boundary constraints to find the best registration output. First the registration is done for k = 4 this results in a single registration for each combination set in k = 4 (~30 000 registrations). The registration transformation matrix associated with the minimum FRE, T_min_ is then used to create the boundary condition of T_min_ ± 3mm (±3pixels shifts for 1 pixel/mm cases). A rigid registration is then computed for each k‐combination set in the k‐set and an FRE is calculated for each rigid registration. This boundary condition is then applied to all calculated registration values to extract the registrations that are within tolerance and their respective k‐combination sets.

The points are then used to find the k‐combination sets for k = 8 and k = 12. CORRO is designed to estimate the optimal registration output for rigid‐registration based on a large set of discrete independent trials, which statistically validate our results using the central limit theorem. The central limit theorem of statistics states that if a large sample is drawn from a population, the distribution of the sample mean is approximately normal and the standard deviation decreases as the number of samples increases.^21^ The larger the set of combination sets, the smaller the registration error which leads to a better estimate of the optimal registration mean of the rigid registration. This statistical theorem has been tested by Castillo et al.,^17^ who developed a framework for evaluating deformable images registration.

### 2.E Estimating the ground truth

**Fig. 3:**
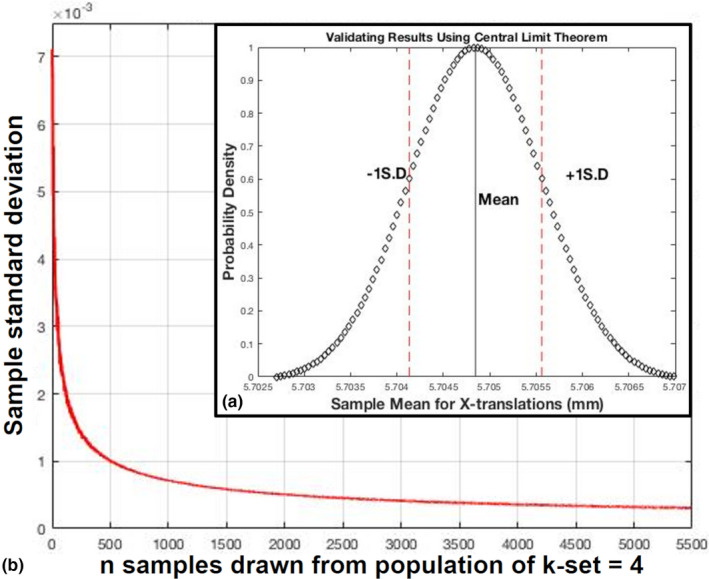
Sample mean distribution for case 3. The distribution showing the x‐translations of k‐combination sets for k = 4.

Having generated a large set of k‐combination sets for each case we are able to estimate the k‐mean of the rigid‐registration and its associated error. The k‐mean is found by calculating the mean of all the translations given by the k‐set registration outputs and the standard deviation (registration error). The results are validated using the central limit theorem evidenced by Fig. 3 for the mean X‐translation for sample case 3.

The k‐set is a large population of sets of paired points, (k‐combination sets) when we draw a large sample from the population of a k‐set the distribution of the sample mean approaches a normal distribution, and the standard deviation of the sample mean decreases as the sample size increases seen in Fig. 3(b). The sampling distribution of the sample mean is found by drawing repeated random samples of a given size n from the population of k‐set for a particular case which has a population mean and a population standard deviation. The mean of all the random samples drawn from the population approximate the population’s mean.

The Gaussian distribution for one case for k‐set of k = 4 is shown in Fig. 3(a) and the standard deviation, which is proportional to $1/\sqrt{n}$, where n is the sample size, is demonstrated in Fig. 3(b). It should be noted that for this particular case the estimated registration (population mean) for about 398 000 k‐set is 5.6816 ± 0.0771 mm for x‐translations and the mean of the sample mean is also 5.7048 ± 0.0718 mm as expected by the central limit theorem.

### 2.F Joint histogram and joint entropy

Information theory is the theoretical foundation on which information content can be quantified.^22^ Entropy measures the randomness or the content of information. Entropy is related to the definition of entropy in thermodynamics, which measures the molecular disorder, or the randomness of a system.

Let *X* be a discrete random variable over χ and probability density function *f* (*x*), *x* ∈ χ. The entropy of *X* is defined as

(6) $$H\left(X\right)=-\sum_{X\in \chi }f\;\left(x\right)\;log\;f\left(x\right)$$

where *f*(*x*) ∈[0,1], $\sum_{x\in \chi }p\left(x\right)=1.0$, and – $\log f\left(x\right)$ is the information associated with a single occurance of *x*. Information is represented in bits and the logarithm is taken in base of 2. Entropy is the function of the distribution of *X*; it does not depend on the actual values taken by the random variable X but only on the probabilities. The expectation value is denoted *E*. Hence, if *X* ~ *f* (*x*), then the expected value of the random variable *g* (*X*) is written as^23^

(7) $$E_{p}g\left(X\right)=\sum_{x\in \chi }g\left(x\right)p\left(x\right)$$

A probability of zero does not contribute to the entropy and as a measure of the average uncertainty in *X*, the entropy is always nonnegative and indicates the number of bits on average required to describe the random variable. The higher the entropy the more information the variable contains. Consequently, the concept of entropy can be extended to two or more variables. The joint entropy of a pair of random variables *H* (*X, Y)* of the **planning CT (*x***) and the registered **CBCT (*y*)** with joint probability distribution is defined as.

(8) $$H\left(X,Y\right)=-\sum_{x\in \chi }\sum_{y\in \Upsilon }f\left(x,y\right)log_{2}f\left(x,y\right)$$

*H* (*X, Y*) is always at least equal to the entropies of *X* and *Y* alone. That is, adding a new variable cannot reduce the existing uncertainty.

The idea of calculating joint entropy for a pair of images is to find the number of possible grey scale values in each image using the 2D joint histogram *f* (*x, y*). If the images are perfectly aligned then the histogram is extremely focused. Misalignment between the images means that there is more dispersion meaning there is higher entropy, as the entropy is the measure of the randomness. In effect images are registered when the coordinate in one image space relates that of the other image to minimize the joint entropy and the randomness is minimized. The opposite is true for unregistered images.

## 3 RESULTS

### 3.A Image Data and landmark pairs

Using Eq. (6) the joint entropy is calculated and a summary of the joint entropy for all six cases is presented in Table 1. The results show that the joint entropy from CORRO is comparable to the results from commercial software.

**Table 1:** Joint entropy of registered images from commercially available software and rigid‐body registered using point combinations of 4, 8, and 12.

Cases	Joint entropy				
	AdaPT insight	MIM	k‐set 4	k‐set 8	k‐set 12
Case 1	5.6906	5.7967	5.7011	5.7165	5.7353
Case 2	5.3351	5.1235	5.0991	5.1034	5.0993
Case 3	6.6121	6.7769	6.6016	6.6184	6.6044
Case 4	8.2827	8.2161	8.3742	8.4288	8.4253
Case 5	4.2473	4.3158	4.2081	4.2391	4.3020
Case 6	4.5894	4.8520	4.5708	4.5622	4.5622

An average of 166 (range: 125–210) landmark pairs were selected for each CT‐CBCT image pair. Table 2 shows the number of landmarks pairs that were selected for each CT‐CBCT case. It should be noted that not every discrete independent combination would give an acceptable k‐set. In this result the same k‐set was reported for k = 4, 8, and 12. Figure 5 shows the combined joint histogram distribution from CORRO (red), AdaPT Insight (Green) and MIM (Blue) for case 3.

**Table 2:** Number of landmark pairs selected for each computed tomography (CT)‐cone beam CT (CBCT) image pair and the total number of k‐combination sets used to estimate the affine fit for each case across all k‐sizes.

Case number	CBCT image dimensions	CT image dimensions	Voxel dimensions (mm)	Number of landmark pairs	Size of k‐Set used to estimate affine fit
1	768 × 768 × 110	768 × 768 × 206	0.6406 × 0.6406 × 2.5	213	80 000
2	768 × 768 × 110	768 × 768 × 185	0.6406 × 0.6406 × 2.5	210	24 000
3	768 × 768 × 110	768 × 768 × 240	0.5176 × 0.5176 × 2.5	141	300 000
4	768 × 768 × 110	768 × 768 × 341	0.5176 × 0.5176 × 2.5	125	55 000
5	768 × 768 × 110	768 × 768 × 173	0.6406 × 0.6406 × 2.5	156	30 000
6	768 × 768 × 110	768 × 768 × 230	0.6406 × 0.6406 × 2.5	211	195 000

### 3.B Trials of Landmark Sets and Rigid‐Body Registration

**Fig. 4:**
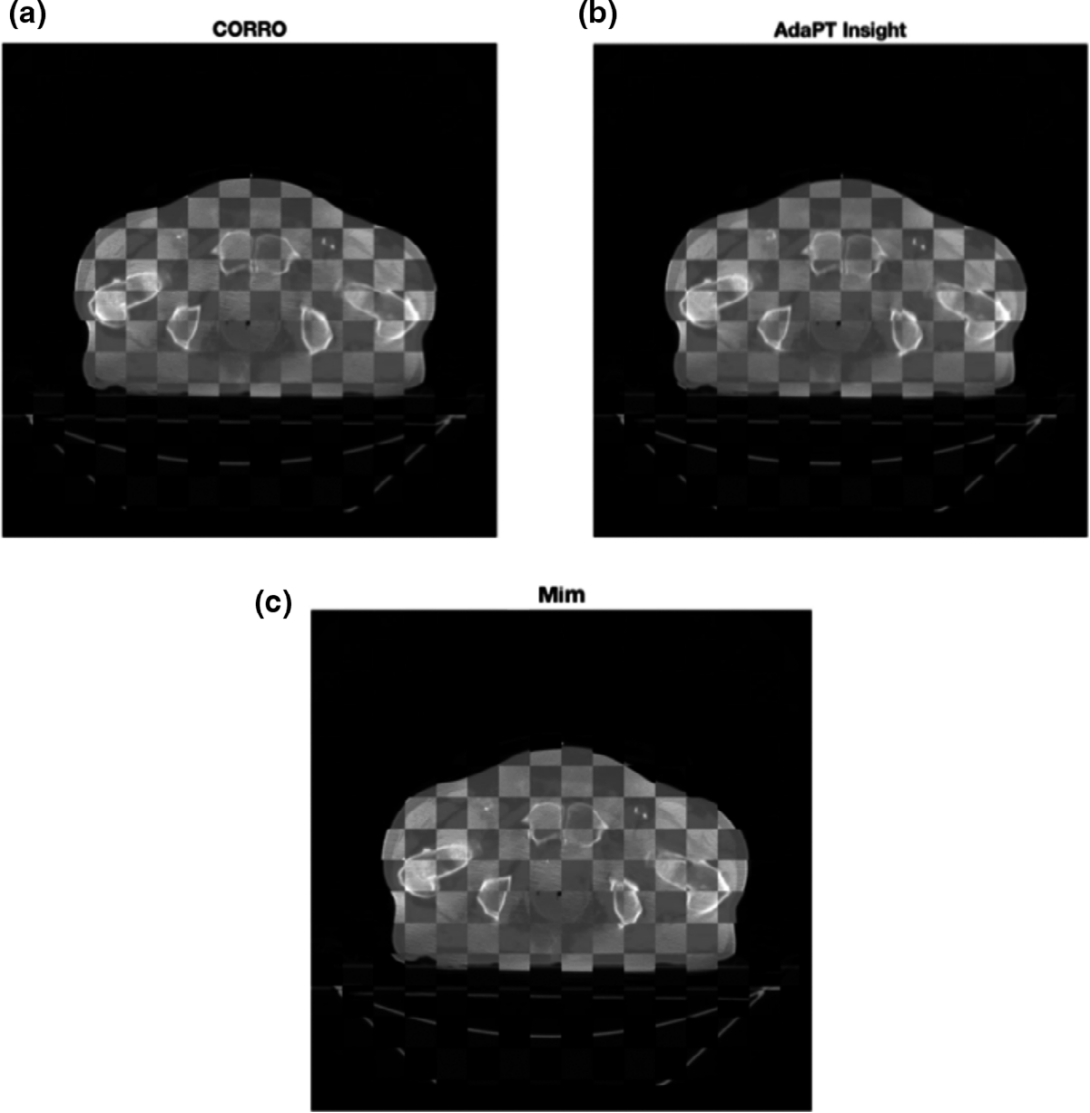
Output registration images for case 2 using checkerboard pattern.

Large k‐sets for k = 4, 8 & 12 were generated and used to solve for the rigid‐body registrations the optimal registration was found by calculating the population mean from the k‐set for a k = 4,8 &12; the standard deviation was found to be the registration error. Table 1 shows the total number of independent trials that were successfully used to perform the registration for each of the cases. Applying the mean registration for combination k = 4 for case 2 to the CBCT the output registration is demonstrated by fusing the planning CT and CBCT to see how well the rigid registration performed. Figure 4 gives a pictorial view of the registration result displayed in a checkerboard form and compared to the registration from the two commercially available registration software packages AdaPT Insight and MIM respectively. Landmark features are well‐aligned in CORRO compared to commercial software.

### 3.C Validating Joint Entropy Results

**Fig. 5:**
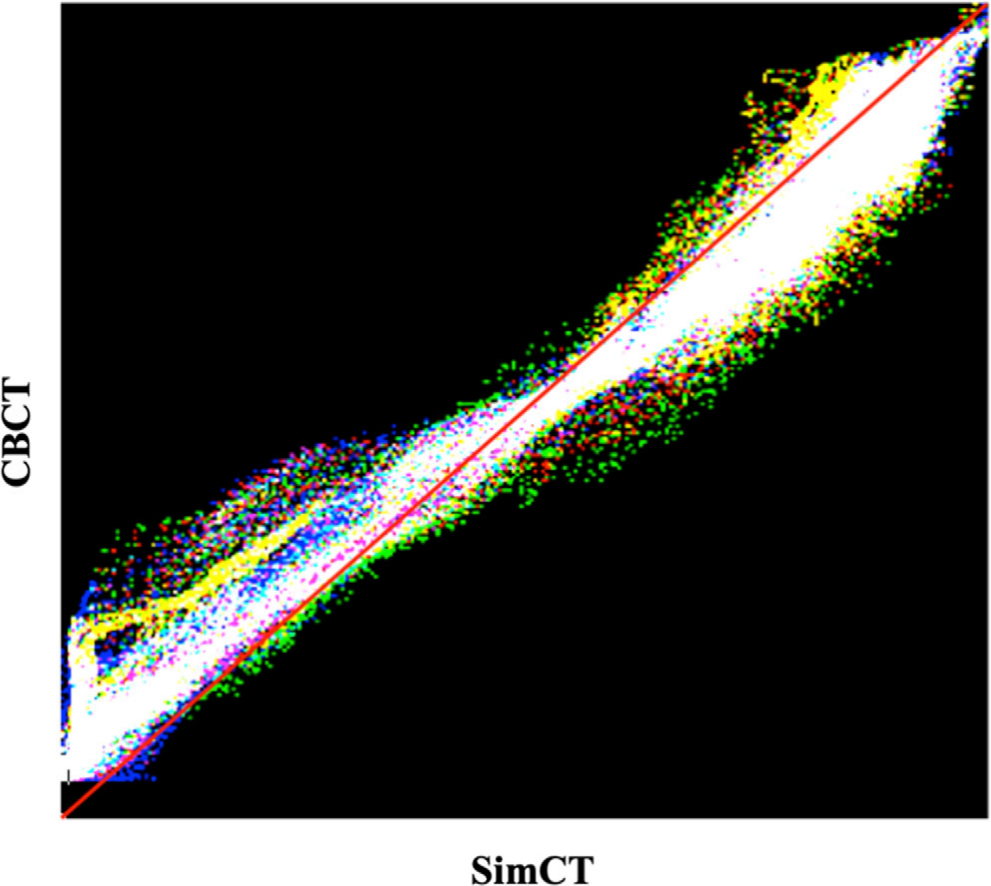
Combination of Joint Histogram distributions from combinatorial rigid registration optimization (Red), AdaPT Insight (Green) and MIM (Blue) for case 3.

Computing the joint entropy of the planning CT and CBCT combination by targeting only the tumor regions for all six cases also validated the results. The joint histogram for all three algorithms CORRO, AdaPT Insight, and MIM were plotted together on Fig. 5, regions of agreement between all joint histograms appear as white. Also, quite a few regions show as yellow, (agreement between CORRO and AdaPT Insight), magenta (agreement between CORRO and MIM), or cyan (agreement between AdaPT Insight and MIM). This can be seen in Table 1 for case 3 where the joint entropy for CORRO and AdaPT Insight are much closer in value than CORRO and MIM or AdaPT Insight and MIM.

**Fig. 6:**
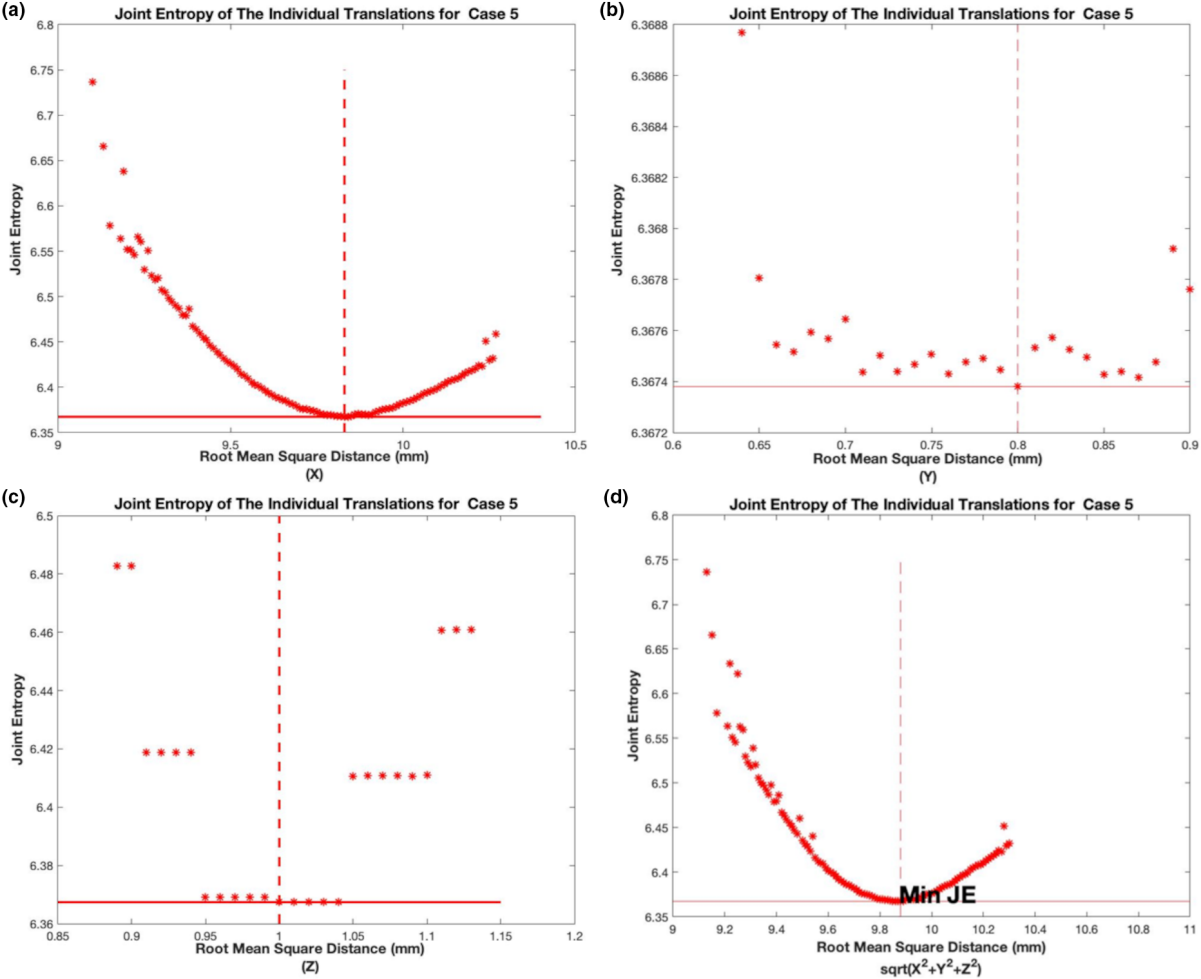
Joint entropy distributions for 30 000 individual transformations Case 5.

The joint entropy for all six cases is tabulated in Table 2 where the results from CORRO are compared to those from AdaPT Insight and MIM software. For all six cases the joint entropy from CORRO were very comparable with very slight differences. Fig. 6 also shows the joint entropy for the 30,000 translations from case 5 for k = 4. The joint entropy for each output was calculated and plotted against the root mean squared distance. The joint entropy is calculated for the image using each member in the k‐set. For example, if there are 190,000 members in the k‐set there will be a total of 190 000 joint entropies calculated. Figure 6 is calculated using 30 000 k‐combination sets the mean translations in the *X, Y, Z* plane are 9.8, 0.76, and 0.96‐mm respectively. When joint entropy was plotted against the *X* translation the minimum joint entropy was found to exist at 9.83 mm in the *X* direction. The minimum joint entropy was found to exist at 0.8 mm pixels when plotted against the *Y* translation and was found to exist at 1.0 mm when plotted against the *Z* translation. Plotting the joint entropy vs the RMS value of *X, Y, Z* translation as seen in Fig. 6(d) the minimum joint entropy was found to exist exactly at the root mean square distances of *X, Y, Z* i.e. $\sqrt{\left(X_{\mathrm{mean}}^{2}+Y_{\mathrm{mean}}^{2}+Z_{\mathrm{mean}}^{2}\right)}$ ^=^ 9.88 mm which is expected.

## 4 DISCUSSION

In order to objectively evaluate clinical results of an image registration algorithm, better attention needs to be given to the selection of appropriate reference points, with which an algorithm performance can be compared. In their work, Wong et al.^24^ investigated the effects of immobilization techniques and organ motion on setup errors. They acknowledged that great care should be taken when developing a clinical database of set‐up errors due to other residual uncertainty factors.

One of the ways in which rigid body registration algorithms have been evaluated is using geometric features. The most widely used of these features in this application are points and surfaces. In this study, we have presented a way of estimating the optimal rigid‐body registration using landmark sets and combinatorial optimization. The estimated optimal ground truth is the mean of all the registration output given by the k‐set. Using expert defined anatomical landmarks and applying the statistical method of combinatorics we generated thousands of landmark point combination sets for each case and estimated the optimal alignment of the rigid registration and the associated error.

The registered images are validated using the root mean squared (RMS) difference of the intensities of the two images, median‐absolute deviation of the intensity difference, and maximum intensity differences.^25^ The results were validated using the central limit theorem and found that, the estimated error associated with rigid registration reduces as we increase the sample size for a particular k‐set as seen in Fig. 3. The results were also validated using joint entropy calculations. A smaller joint entropy value shows there is less variation in the pixel intensities and the similarity between the images is high implying a better registration. Joint Entropy is a common method employed to validate the quality of radiographic images, analyze the similarity of images, and also validate registration output between multimodal image registrations.^26^, ^27^

The registration results from CORRO using the mean as the optimal registration were compared to commercially available registration software using joint histogram and joint entropy analysis. A comparison between the joint entropy values of the rigid registration and those from the commercially available software were very closely related as seen in Table 2. The results of k = 4 gave lower joint entropy values when compared to k = 8 and 12 and the commercial software indicating slightly better registration results using k = 4. This was not surprising because k = 8 and 12 are more likely to have mismatched points that would affect the accuracy of the registration.^28^ In 3D any four points can be mapped to another four points.^29^ However, more landmarks result in transformations with nonuniform biased sampling of the image. Coste^30^ shows that using many landmarks could result in odd transformations of the image grid. Our data comparing CORRO for k = 4 and k = 8 and k = 12 seem to support such a hypothesis k = 4 demonstrates superior registration results in comparison to k = 8 and 12. Hence k = 4 can be best used to determine the optimal registration results.

Lastly, the joint entropy for all the translations plotted against the root mean square (RMS) distances of the output translations (X, Y, Z). The root mean square distance is given by $\sqrt{Xmean^{2}+Ymean^{2}+Zmean^{2}}$. We found the minimum joint entropy value to be exactly equal to the RMS value. Signifying that the mean translation in *X, Y, Z* are equivalent to the optimal rigid registration translation outputs as determined by the calculated minimum joint entropy. It should be noted that the landmark selection process is time consuming. Hence this study will not be suitable for clinical routine. This work was carried out to demonstrate the feasibility of finding the optimal rigid transformation using large sets of landmark points; in the future automated techniques could be used to identify the landmark points.

## 5 CONCLUSION

The results of this study show that we have developed a methodology called CORRO that allows us to estimate optimal alignment for rigid‐registration of clinical image pairs using a large set of discrete independent trials of large landmark points combination sets. The viability of this method has been demonstrated with six cases: two pelvic, two head and neck, and two thoracic. The results for the rigid‐body registration have been shown to be comparable to results from commercially available algorithms for all six cases. CORRO can serve as an excellent metric for registration quality assessment. The next step in this project is using CORRO to create a statistically characterized reference data set for 58 pelvic cases that are being made available at the cancer imaging archives (https://wiki.cancerimagingarchive.net/display/Public/Pelvic+Reference+Data).

## CONFLICT OF INTEREST

Authors declare no financial relationships that may lead to a conflict of interest.
